# Pattern of intracranial findings detected on magnetic resonance imaging in surviving infants born before 29 weeks of gestation

**DOI:** 10.1371/journal.pone.0214683

**Published:** 2019-04-04

**Authors:** Anna Petrova, Sreenivas Reddy, Rajeev Mehta

**Affiliations:** 1 Department of Pediatrics, Rutgers Robert Wood Johnson Medical School, New Brunswick, New Jersey, United States of America; 2 School of Biomedical Sciences, Rutgers the State University of New Jersey, Piscataway, New Jersey, United States of America; Wayne State University, UNITED STATES

## Abstract

Despite the positive survival trend in infants born prematurely, the risk for development of intracranial lesions has remained unchanged. However, there are limitations to our understanding of the pattern of the magnetic resonance imaging (MRI) -detected brain pathology in the preterm infants surviving to discharge. The present study outlines the type of intracranial lesions and factors allied with the neonatal brain hemorrhage (NBH) and white matter injury (WMI) seen on MRI at term-equivalent age or close to discharge in infants born before 29 weeks of gestation. We obtained demographic and clinical data, and reports of serial cranial ultrasound (CUS) performed during first month of life and qualitative MRI at term-equivalent age or close to discharge. Statistical comparison was conducted with respect to the MRI results that were classified as normal, WMI, and NBH using univariate and logistic regression analysis. One hundred and ninety three infants with MRI at term-equivalent age or close to discharge were included in final analysis. They were less mature and had a higher prevalence of pathological findings on CUS as compared with 249 other survivors born with gestational ages less than 29 weeks during the assigned study period. MRI was normal in 72.5% [95% Confidence Interval (95% CI 65.9%-78.4%)], showed WMI in 9.8% (95%CI 6.4%-14.9%) and NBH in 17.6% (95%CI 12.9–23.6) of the studied infants. Intracranial hemorrhages had also been reported in 42.2% of the infants with WMI. Except for moderate agreement with prior CUS results, no other factors were associated with the MRI detected pathological findings. In general, the likelihood for detection of WMI and NBH on MRI at term-equivalent age or close to discharge was reduced by approximately 80% and 70%, respectively if the serial CUS had not shown any abnormalities during the first month of life.

## Introduction

A large body of evidence demonstrates an encouraging survival trend in prematurely born infants [1, 2]. However, the risk for development of intracranial lesions that are mainly associated with the vulnerability of the immature brain to oxidative stress, hemodynamic instability and inflammation, has remained unchanged [3, 4]. The key role of intracranial injury in the development of serious neuropathology in prematurely born infants is a noticeable public health concern related to the consequences of preterm delivery [5,6]. With that in mind, serial cranial ultrasound (CUS) imaging was initiated to ensure surveillance of intracranial findings in very preterm born infants during their birth hospitalization [7,8]. Magnetic resonance imaging (MRI) was also suggested for the precise detection of white matter injury (WMI) [9–12] because the contribution of WMI to the development of preterm-birth associated neuropathology is fully acknowledged [13–15]. Periventricular leukomalacia (PVL) and other WMI such as encephalomalacia, hemorrhage in white matter, post-hemorrhagic ventriculomegaly, ventricular dilation, porencephaly, and altered cerebral volume, contribute significantly to the development of long-term neurodevelopmental deficits in premature infants [13,15–18]. Neonatal brain hemorrhage (NBH) including intraventricular hemorrhage (IVH) and germinal matrix hemorrhage also contribute to the neurological morbidities seen in prematurely born infants [19–21]. Despite the relevance of the neuroimaging findings in the development of long-term neuropathology, the pattern of MRI-detected lesions in surviving preterm neonates is still the subject of discussion. The literature on this topic is limited to the several reports that vary in the gestational age of the study participants and classification of the findings detected on pre-discharge MRI [16,22,23].

This study was primarily designed to evaluate the pattern of the MRI-detected intracranial hemorrhages and white matter alterations reported in surviving neonates born at a gestational age of less than 29 weeks. We aimed to assess not only the prevalence of WMI and NBH, but also the associated factors and agreement between the data detected on serial CUS and MRI performed at term-equivalent age or close to discharge. The results of this study may contribute to a better understanding of the causal pathways of the intracranial lesions diagnosed in surviving infants born at less than 29 weeks gestation, and therefore, may help in the creation of preventive strategies to reduce the burden of prematurity-related long-term neuropathology.

## Materials and methods

The study (Pro 20150001473) was approved by the Institutional Review Board at Rutgers Robert Wood Johnson Medical School without any parental consent being required.

We used the electronic medical records (EMR) to identify neonates born at less than 29 weeks gestational age and admitted to the level III Neonatal Intensive Care Unit (NICU) at Bristol-Myers Squibb Children’s Hospital—Robert Wood Johnson University Hospital (RWJUH) between January 1, 2004 and December 31, 2014. A standardized tool was constructed to collect the demographic, clinical and neuroimaging data on all the preterm infants born prior to 29 weeks gestation and hospitalized during the study period. The collected maternal data included parity (nuliparity vs. multiparity), type of pregnancy (singleton vs. multiple), mode of delivery (cesarean vs. vaginal), preterm premature rupture of membranes (PPROM), clinical chorioamnionitis, pre-eclampsia, fetal distress, and placental pathology (abruption, previa). Information regarding the administration of intrapartum corticosteroids and magnesium sulfate (MgSO_4_) whether for tocolysis/neuroprotection/pre-eclampsia treatment was also collected. The neonatal data that was collected included gestational age (GA) in completed weeks, birth weight (BW), Apgar scores at 1 and 5 minutes, and neonatal morbidities such as echocardiography-detected patent ductus arteriosus (PDA), clinical sepsis, necrotizing enterocolitis (NEC), seizures, apnea of prematurity, and severe thrombocytopenia (<50,000/mL).

### Analysis of the neuroimaging data

We reviewed the neuroimaging reports from sequential CUS performed within the first week and at 3–4 weeks of post-natal age, and MRI that was completed at term-equivalent age or close to discharge. The IVH seen on CUS was reported as grade I (hemorrhage isolated to the germinal matrix), grade II (with intraventricular extension but without ventricular enlargement), grade III (with intraventricular extension and ventricular enlargement), and grade IV (with intraventricular and intraparenchymal involvement) [24]. CUS detected localized or diffuse periventricular echolucency was classified as cystic or no cystic PVL [11]. MRI data were acquired using a 1.5 Tesla General Electric Excite system (GE Medical Systems, Milwaukee, WI) and the same imaging protocol for all patients during the 10 year study period. The protocol includes: axial diffusion weighted imaging (DWI), sagittal short inversion time inversion recovery (STIR), axial T1 sequence, axial gradient echo sequences (Axial GRE), axial T2-weighted propeller, axial T2 fluid attenuation inversion recovery (FLAIR), and apparent diffusion coefficient (ADC). MRI outlines the structural alterations of the white matter, hemorrhages, and ventricular size based on configuration of T1 and T2 intensities:(i) T1 hypointensities and T2 hyperintensities to identify the undermyelination, demyelination, neuronal or axonal loss and gliosis, and (ii) T1 hyperintensities and T2 hypointensities, and T2* hypointensities from gradient echo to detect hemorrhage, and mineralization. Signal intensity changes detect the structural alterations in the white matter, such as cysts, cavitations, and atrophy as well as hemorrhages in germinal matrix and IVH, and post-hemorrhagic hydrocephalus [25]. Based on the above-mentioned techniques, the MRI findings were reported as either normal or showing a variety of lesions in the white matter (WMI) or neonatal brain hemorrhage (NBH). Moreover, the MRI reports provided to the clinicians included a comparison with prior CUS studies and identified the stage of hemorrhage and whether the IVH had resolved, remains unchanged, or residual.

### Definition of the study groups

The current report includes an analysis of infants born at less than 29 weeks of gestation, who survived to discharge and underwent a cranial MRI at close to a term equivalent age or prior to discharge as ordered by the attending neonatologist. The infants were grouped based on their MRI findings. A review of the MRI reports revealed the variability of findings, including no abnormality (normal), lesions in the white matter (PVL, encephalomalacia, porencephaly, altered cerebral volume, hemorrhage in the white matter, and post-hemorrhagic ventricular dilatation), and hemorrhages at different stages (resolved, residual, unchanged) in the ventricles and germinal matrix. Given the small number of children with different abnormalities in white matter, those with any WMI were combined for the purpose of this analysis. We summarized the MRI reported findings into three groups: Normal, WMI, and NBH. Infants diagnosed with WMI and hemorrhages in the germinal matrix/IVH were included in the WMI group. The NBH group comprised infants with hemorrhage in the germinal matrix/IVH at residual or unchanged stages without evidence of involvement of the white matter. The normal MRI group included infants with either no pathological findings or resolved old hemorrhages without any post-hemorrhagic ventricular dilatation/ventriculomegaly/hydrocephaly.

#### Statistical analysis

We compared the demographic and clinical maternal and neonatal data as well as serial CUS results with respect to the MRI findings at term-equivalent age or close to discharge using Chi-square and analysis of variance (ANOVA), followed if required, by Tukey’s honest significance difference test. After grouping the infants depending on their MRI findings, stepwise binary logistic regressions were performed to evaluate which factors were associated with the diagnosis of WMI and NBH in the studied infants. Infants with normal MRI were used as a reference group. In unadjusted univariate analysis, the predictive variables for inclusion into models with P- value of less than 0.1 were selected. Gestational age at birth was included in the models irrespective of the significance due to importance of gestational age in the development of brain injury in preterm born infants. The clinical and CUS data were classified and coded as binary variables defined as “yes pathology” (coded 1) versus “normal” (coded 0). Low grade IVH detected on CUS was coded 1 because IVH grade 1 was found to be associated with an almost two-fold higher risk for the diagnosis of prematurity-associated neurosensory impairment [21]. Data are presented as mean, percentage (%), and Odds Ratios (OR) with 95% Confidence Interval (95%CI). We also evaluated agreement of the MRI-detected lesions with serial CUS performed within the first week and 3–4 weeks of postnatal age. The kappa coefficient (k) and 95% Confidence Interval (95%CI) of (k) was calculated taking into account any random chance for agreement between the CUS and MRI reports. The results were classified as disagreement (less than 0), no agreement (0–0.20), minimal (0.21–0.39), weak (0.40–0.59), moderate (0.60 to 0.79), strong (0.80 to 0.90), and almost perfect (above 0.90) agreement [26]. STATISTICA 13.2 (StatSoft, Tulsa, OK, USA) was used to analyze the study results. P value of less than 0.05 was considered to indicate a statistically significant difference.

## Results

Medical records of 527 preterm infants born at a gestational age of less than 29 weeks were reviewed. Eighty-eight (16.7%) infants died before discharge. Among the 439 survivors, 193 infants with MRI performed at term-equivalent age or close to discharge were entered into the final analysis. These infants were born with lower gestational age, birth weight, and Apgar scores, and were more frequently diagnosed with intracranial lesions on serial CUS than infants with no MRI performed at term-equivalent age or close to discharge (Table 1).

**Table 1:** Comparison of patients with and without MRI performed at term-equivalent age or close to discharge.

Patient characteristics	MRI done			
	Yes (n = 193)		No (n = 229)	
Gestational age (weeks)*	25.8 (25.6, 26.0)		26.5 (26.3, 26.7) ã	
Birth Weight (grams)*	899 (864, 934)		920 (882, 953)	
Apgar 1 min (score)*	4.2 (3.9, 4.5)		5.3 (5.0, 5.6) ã	
Apgar 5 min (score)*	6.5 (6.3, 6.8)		7.1 (6.9, 7.3) ã	
**CUS**	**Within 7 days** **	**3–4 weeks** ***	**Within 7 days** **	**3–4 weeks** ***
NormalIVH G1IVH G2IVH G3IVH G4PVL	61.4% 18.7% 8.3% 5.2% 6.2% 0.5%	65.8%16.6%3.1%7.3%5.7%1.6%	79.9% 11.4%3.1% 2.6%3.1%0	79.5%12.2%1.8%2.6%3.9%0

* Mean with (95% Confidence Interval)

γ Difference (P<0.001) between patients with and without pre-discharge MRI

**Difference (P<0.001) between pathological findings on CUS performed within 7 days in infants with and without pre-discharged MRI

***Difference (P<0.001) between pathological findings on CUS performed on 3–4 weeks after birth in infants with and without pre-discharged MRI

### Description of MRI-detected findings

Among the 193 studied infants, the MRI identified no pathology (normal) in 140 [72.5%, (95%CI 65.9%-78.4%)] and WMI/NBH in 53 [27.5% (95%CI 21.7%, 34.2%)], which included 19 [9.8%, (95%CI 6.4%-14.9%)] with WMI and 34 [17.6% (95%CI 12.9–23.6)] with NBH. The WMI group included infants with PVL (n = 6), hemorrhage in white matter (n = 4), encepalomalacia (n = 3), loss of white matter volume (n = 2), porencephaly (n = 1), and post-hemorrhagic ventricular dilation (n = 3). Two patients had cystic lesions in the white matter. Hemorrhage in the germinal matrix/IVH was recorded in 8 (42.1%) of the patients included in the WMI group. A few infants in the NBH group had been diagnosed with post-hemorrhagic encephalomegalia (n = 1) and ventricularmegalia (n = 1). Among the infants in the normal group, four were diagnosed with benign external hydrocephalus without enlargement of ventricles, and no follow up was recommended by the neuroradiologist.

### Factors associated with the MRI detected intracranial lesions

**Fig 1:**
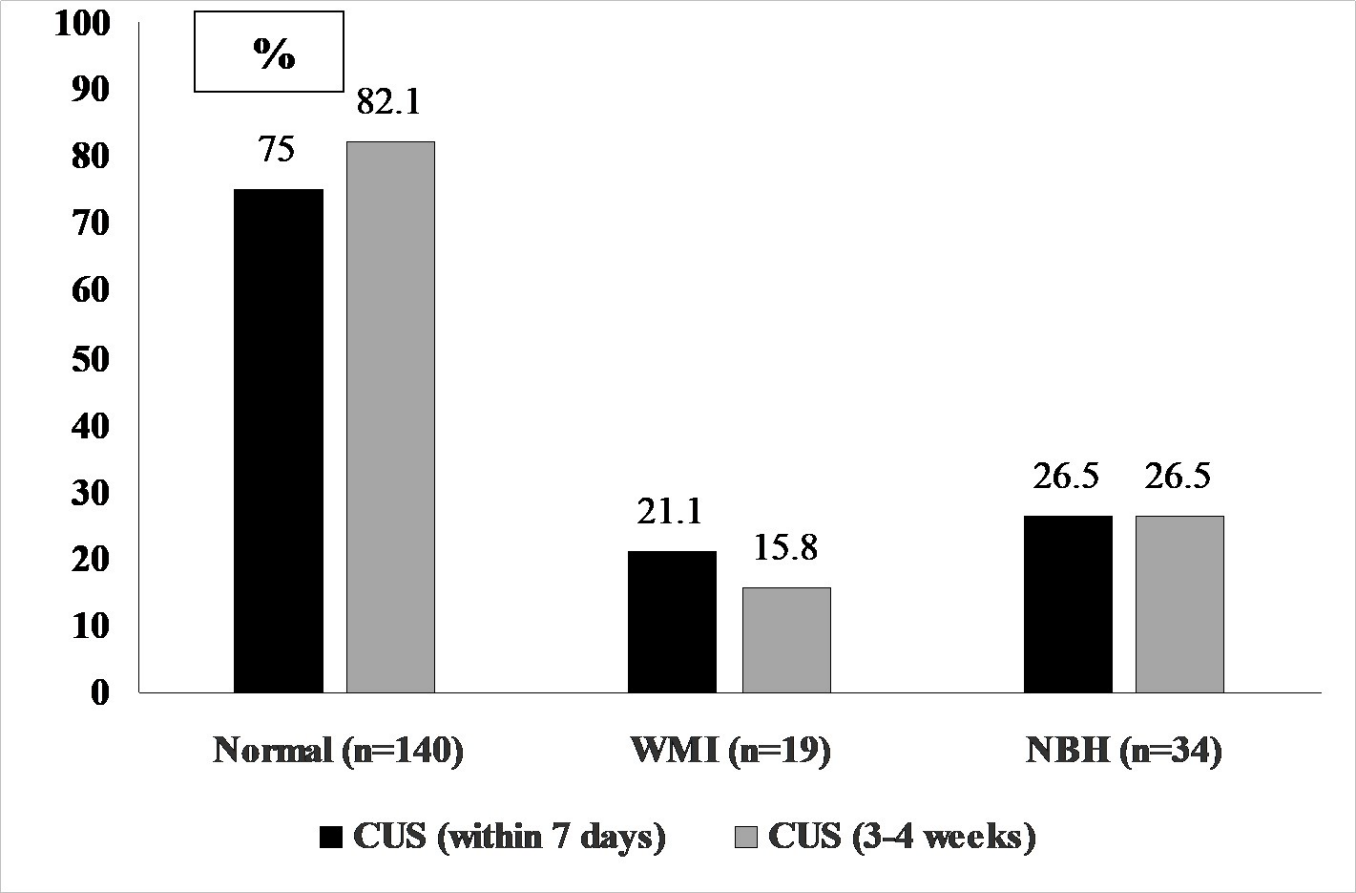
Proportion of normal CUS reports with respect to the MRI detected findings.

**Fig 2:**
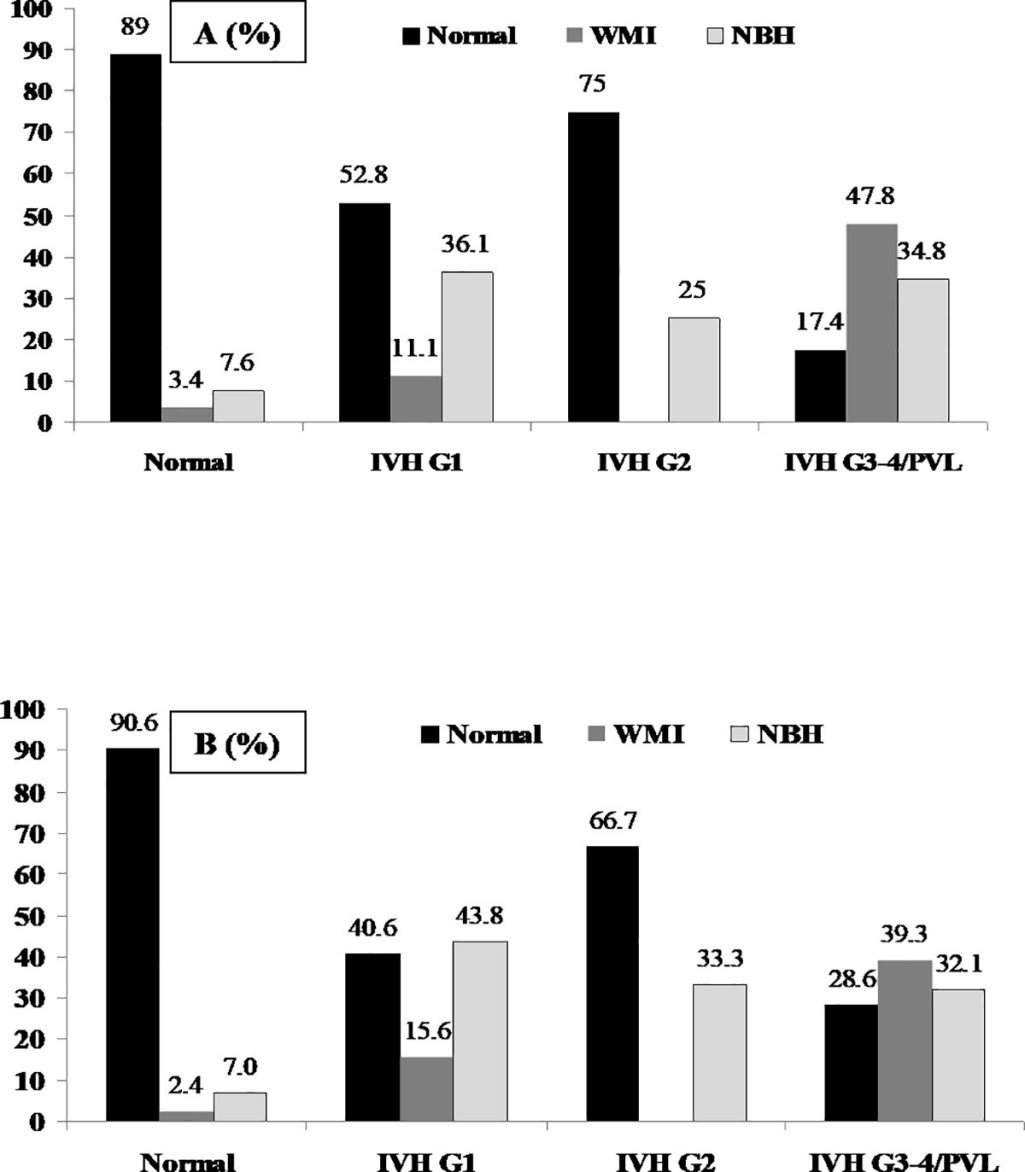
MRI detected intracranial findings with respect to the results of the CUS performed within 7 days (A) and at 3–4 weeks of post-natal age (B).

As shown in Table 2, the characteristics of the infants categorized as “with” and “without MRI-detected intracranial lesions” at term-equivalent age or close to discharge were comparable except for placental pathology that was diagnosed more frequently in infants with WMI. Overall, approximately 80% of patients with a normal MRI, 20% with white matter lesions (WMI), and 26% with neonatal brain hemorrhages (NBH) had not shown any pathology on the serial CUS during the first month of life (Fig 1). We also compared the MRI results with the type of brain pathology detected by CUS in the first 7 days of life (Fig 2A) and at 3–4 weeks of post-natal age (Fig 2B). The MRI was normal in 52.8% and 40.6% of the infants diagnosed with IVH Grade 1 by CUS in the first 7 days and at 3–4 weeks of life, respectively (P<0.02). Among patient with IVH Grade 2 diagnosed by serial CUS, 75% (Fig 2A) and 66.7% (Fig 2B) respectively, showed no pathology on MRI at term-equivalent age or close to discharge (P = 0.08). The MRI was normal in 17.4% and 28.6% (P<0.01) of infants previously diagnosed with IVH Grade 3-4/PVL by the two consecutive CUS (Fig 2A and 2B). We did not find a significant difference in the MRI-detected WMI (47.8% vs. 39.3%, P = 0.09) and NBH (34.8% vs. 32.1%, P = 0.57) in infants diagnosed with Grade 3-4/PVL by CUS in the first 7 days and at 3–4 weeks of life, respectively. Irrespective of the gestational age and placental pathology, data from the regression analysis confirmed a significant risk reduction for infants to be diagnosed with WMI and NBH by the MRI at term-equivalent age or close to discharge if the CUS performed during their hospital stay had not detected any abnormal findings (Table 3). The MRI at term-equivalent age or close to discharge showed only minimal overall agreement with CUS performed within seven days after birth (k 0.45, 95% CI 0.31, 0.58) whereas when the MRI results were compared with CUS performed at 7 days and 3–4 weeks of life, respectively, there was moderate overall agreement (k 0.55, 95%CI 0.42, 0.68). Disagreement between the results of the CUS at 3–4 weeks, which were categorized as normal vs. IVH/PVL and findings on MRI at term-equivalent age or close to discharge, which were categorized as normal vs. WMI/NBH, was recorded in 18.7% (36/193) of the studied infants. Infants showing disagreement between the CUS and MRI were more likely to have been born with gestational age less than 27 weeks as compared to those with consistent neuroimaging findings (83.3% vs.63.4%), P<0.03. No other factors were found to be significantly associated with the recorded disagreement between the CUS and MRI findings (data not presented). Among 36 patients, 25 (69.4%) with normal CUS did not show any abnormality in their MRI and 11 patients (30.6%) with normal CUS were found to have WMI/NBH on MRI at term-equivalent age or close to discharge.

**Table 2:** Comparison of maternal and neonatal characteristics of the studied infants.

Characteristics	MRI findings			P value
	No pathology (n = 140)	WMI (n = 19)	NBH (n = 34)	
Gestational age at birth, wk	25.8+/-1.5	25.3+/-1.2	26.1+/-1.5	0.25
Singleton pregnancy, % (n)	80.0 (112)	94.7 (18)	82.4 (28)	0.49
Nuliparity, % (n)	52.2 (71)	61.1 (11)	58.8 (20)	0.64
Preeclampsia, % (n)	18.6 (26)	15.8 (3)	14.7(5)	0.73
Fetal distress, % (n)	5.0 (7)	10.5 (2)	5.9 (2)	0.62
PPROM, % (n)	29.3 (41)	31.6 (6)	35.3 (12)	0.78
Antenatal MgSO4, % (n)	40.7 (57)	36.8 (7)	38.2 (13)	0.93
Antenatal corticosteroids, % (n)	65.7 (92)	73.7 (14)	79.4 (27)	0.69
Placental complications, % (n)	13.6 (19)	31.6 (6)	5.8 (2)	0.03
Chorioamnionitis, % (n)	8.6 (12)	10.5 (2)	8.8 (3)	0.96
Cesarean Section, % (n)	74.3 (104)	68.4 (13)	58.8 (20)	0.19
Male gender, %(n)	42.1 (59)	52.6 (10)	44.1 (15)	0.68
Birth weight (g)	892+/-226	872+/-269	947+/-263	0.24
Apgar 1 min (score)	4.3+/-2.1	3.9+/-2.6	4.1+/-2.5	0.55
Apgar 5 min (score)	6.6+/-1.8	6.3+/-1.6	6.3+/-2.0	0.55
Surfactant administration, % (n)	83.7 (113)	93.3 (14)	88.2 (30)	0.52
Thrombocytopenia,% (n)	7.3 (11)	0% (0)	9.1 (3)	0.44
Patent ductus arteriosus, % (n)	38.1 (53)	44.4 (8)	32.4 (11)	0.67
Sepsis, % (n)	38.9 (54)	38.9 (7)	41.2 (14)	0.96
Necrotizing enterocolitis, % (n)	5.0 (7)	5.6 (1)	0% (0)	0.44
Seizures, %(n)	1.4 (2)	5.3 (1)	8.8 (3)	0.07
Apnea prematurity, % (n)	30.3 (43)	33.3 (6)	32.4 (11)	0.83

Plus-minus values are mean with standard deviation (+/-SD)

**Table 3:** Predictive models for MRI detected WMI and NBH (OR, 95%CI)*.

Factors	MRI-detected pathology	
	WMI	NBH
Gestational age at birth (weeks)	0.81(0.69, 1.13)	1.13 (0.87, 1.46)
Placental complications**	1.71 (1.0, 2.98)	1.49 (0.69, 3.17)
Normal** on CUS (within 7 day)	0.28 (0.16, 0.94)	0.35 (0.23, 0.53)
Normal** on CUS (3–4 week)	0.20 (0.11, 0.39)	0.28 (0.18,0.43)

* Compared to infants with no pathology (normal), coded 0

** Yes pathology vs. normal, coded 1 and 0, respectively

## Discussion

In our study, qualitative analysis of the MRI of the brain identified WMI and NBH in approximately one-third of the surviving infants born before 29 weeks of gestation. The estimated population-based prevalence of NBH was 1.5 times higher than WMI. Intracranial hemorrhages were also co-diagnosed in 42% of cases with WMI, which is consistent with the known role of NBH in the development of WMI in prematurely born infants [27,28]. In preterm infants who had no evidence of intracranial lesions on serial CUS performed during the first month of life, there was almost 80% reduction in risk for discharge with WMI or NBH. Nevertheless, the agreement between the serial CUS and MRI was mild to moderate perhaps due to the limited ability of CUS to characterize the wide spectrum of cerebral abnormalities in preterm neonates [11,29,30]. Comparison of the existing reports regarding the pattern of MRI findings in surviving preterm born infants is limited, mainly due to the variability of the MRI reporting system used in relevant research. Inconsistency in the configuration of MRI detected intracranial lesions in preterm born infants surviving to discharge has been reported [31]. Qualitative analysis of the MRI findings showed punctate WMI in 33% and IVH in 24% of extremely preterm born infants [32]. Quantitative analysis of MRI identified severe brain injuries in 10% of infants born with a gestational age of less than 30 weeks [22] and moderate to severe WMI in 5.9% to 35.0% of extremely preterm infants from different settings [16]. Among infants born at a gestational age of less than 28 weeks, 20% [11] and 17% [23] were diagnosed with moderate to severe WMI; however, different criteria had been used to score the MRI findings. Gano et al [33] reported moderate/severe WMI on MRI within 4 weeks of birth in 17% of very preterm born infants. Quantitative analysis of the non-hemorrhagic WMI identified cystic WMI in 21 infants born at a gestational age of 35 weeks or less among the 42 who underwent MRI at term equivalent age [10].

We would like to acknowledge several limitations of the present study. First, the utilization of clinical data collected not for a research purpose could be the source of selection and misclassification bias. However, EMR files are increasingly being seen as a valuable resource for research [34, 35]. The data used in our study were in keeping with the definition of research quality [36]: (i) the utilization of objectively collected data for the selection of study subjects to answer the research question (gestational age and brain MRI at term), and (ii) uniformity of data collection that was accomplished with the standardization of the data extraction tool. Second, the results of our study are not applicable to all preterm infants born with a gestational age less than 29 weeks but only to those who are at highest risk for developing cerebral alterations due to the level of their immaturity (Table 1). Third, qualitative MRI reports could be less informative than those based on a quantitative scoring system [37]. Although an association between score severity and prediction of preterm neuropathology has been reported [13], the pattern between MRI detected severities of cerebral white matter abnormalities and neurodevelopmental outcome is questionable [16, 38]. Global brain abnormality score of MRI detected injuries in preterm born infants has not been widely implemented as yet in radiological practices. There is a lack of consensus regarding how definitely to interpret and utilize MRI results in order to maximize the practical value of MRI testing in clinical neonatology because the MRI at term in infants born prematurely has not been formally standardized in the clinical settings [39, 40]. Without standardization of the MRI reports, the effectiveness of utilization of MRI at term in both, the clinical management and research of preterm born infant is limited. Moderate/severe WMI seen on MRI around term can predict cerebral palsy and motor function with moderate sensitivity and specificity and has limited ability to predict neurocognitive and behavioral impairments [41]. Moreover, diffuse excessive high signal intensity (DEHSI) that is included in the scoring system has demonstrated limited prognostic value. Importantly, a recent large scale randomized clinical trial identified that as compared to CUS, MRI at term increases costs and provides only a modest benefit related to the reduction of maternal anxiety and prediction of the infant’s neurodevelopment outcome [42]. It is obvious that increased use of MRI at term-equivalent age or close to discharge will require implementation of consistent evaluation of intracranial findings in preterm born neonates [16].

### Conclusions

Qualitatively assessed configuration of intracranial injuries detected on MRI at term revealed the risk for injuries in white matter in up to 15% and isolated hemorrhagic lesions in up to 24% of less mature survivors born before 29 weeks of gestation. Hemorrhages of any grade that are detected on serial CUS should be taken into account for more precise prediction of the intracranial lesions seen on MRI performed at the time of discharge.

## Supporting information

S1 Dataset (XLSX)
